# Quantitative Analysis of Phenolic Acids and Flavonoids in *Cuscuta chinensis* Lam. by Synchronous Ultrasonic-Assisted Extraction with Response Surface Methodology

**DOI:** 10.1155/2018/6796720

**Published:** 2018-12-20

**Authors:** Kun-ze Du, Jin Li, Xinrong Guo, Yuhong Li, Yan-xu Chang

**Affiliations:** ^1^Tianjin State Key Laboratory of Modern Chinese Medicine, Tianjin University of Traditional Chinese Medicine, Tianjin 300193, China; ^2^Tianjin Key Laboratory of Phytochemistry and Pharmaceutical Analysis, Tianjin University of Traditional Chinese Medicine, Tianjin 300193, China

## Abstract

An effective ultrasonic-assisted extraction method for the separation of phenolic acids and flavonoids in *Cuscuta chinensis* Lam. was conducted by combining uniform design (UD) coupled with response surface methodology (RSM) and orthogonal design (OD) experiment. A sensitive and selective high-performance liquid chromatography-electrospray ionization tandem triple quadrupole mass spectrometry (HPLC-ESI-MS/MS) method was applied to quantify the sixteen active ingredients (chlorogenic acid, cryptochlorogenic acid, neochlorogenic acid, isochlorogenic acid A, isochlorogenic acid B, isochlorogenic acid C, caffeic acid, hyperin, isoquercitrin, quercetin, campherol, *p*-coumaric acid, isorhamnetin, rutin, astragalin, and apigenin). The extraction method was optimized with respect to concentration of extraction solvent, extraction time, and ratio of liquid to solid as a consequence of getting a high sensitive and feasible method for simultaneous determination of contents of multiple components and evaluation of quality control of *Cuscuta chinensis* Lam. from different origins. It was also considered useful and valuable in the further study for quality control of *Cuscuta chinensis* Lam.

## 1. Introduction

Traditional Chinese medicines (TCMs) occupy incomparable position in the pharmaceutical industry due to their extensive activity for preventing and treating diseases. Because of their clinic application and the contribution for drug discovery, TCMs have been drawn widespread attention in the world [1]. Only one or very limited kind of constituent was stipulated as a marker for the quality control of herb according to the authoritative Chinese Pharmacopoeia 2015. However, hundreds of constituents could be extracted from a single herb, which may exert various pharmacological functions and diverse bioactivities [2]. On account of the intimate connection with pharmacological activity, the quality of TCMs is increasingly strict and more components in TCMs should be identified and quantified grimly.

As a commonly used TCM, cuscutae semen, dried fruits of *Cuscuta chinensis* Lam., is widely distributed in China. This annual parasitic herb is parasitic on the legume, compositae, chenopodiaceae, and other herbs commonly, such as *Artemisia lavandulaefolia* DC, *Lespedeza chinensis* G. Don, and *Vicia cracca*. It has been employed in various clinical applications, including female infertility, preventing abortion, male reproductive system disease, chyluria, and chloasma faciei. [3] The extensive modern pharmacological studies have indicated that *Cuscuta chinensis* Lam. could decrease the apoptosis of cardiomyocytes [4], exhibit antifibrotic effect [5], suppress the inflammatory response [6], improve sexual potency, prevent abortion, and enhance liver and kidney conditions [3]. Phytochemical compounds of *Cuscuta chinensis* Lam. incorporate flavonoids, phenolic acids, volatile oils, hydroquinones, lignans, fatty acids, resin glycosides, steroids, polysaccharides, and alkaloids. By reason of the highest proportion in the chemical components of *Cuscuta chinensis* Lam., flavonoids and phenolic acids are two kinds of major bioactive compounds isolated from this TCM. A few results of studies suggested that some *Cuscuta chinensis* Lam. extracts could exert various pharmacological activities, for instance, flavonoids, phenolic acids, and phenolic compounds acting as free-radical scavengers could be responsible for antioxidative activity [3, 7].

A few analytical methods of quality control for *Cuscuta chinensis* Lam. were applied, such as HPLC-UV [8–10], high-performance capillary electrophoresis (HPCE) [11], and thin-layer chromatography (TLC) [12]. However, these practical methods either have very low sensitivities or were simply applied for evaluating limited kinds of active constituents such as flavonoids and polysaccharides. Due to the characteristics of multicomponent and multitarget of TCMs, it is difficult to comprehensively reveal the quality of TCMs via determining the few compounds merely. Therefore, it is necessary to establish a more precise analytical method of determining multiple compounds for assessing the quality of TCMs.

More useful information and optimum experimental conditions could be achieved by a good design and suitable model of experiment. Recently, uniform design (UD), which was shown to be a promising experimental design method and more effective than orthogonal design (OD) relying on numerical experiments, was reportedly used in the field of chemometrics, sciences, pharmaceutics, engineering, and manufacturing [13–18]. Response surface methodology (RSM) could be used to improve and optimize the complex experimental processes as a collection of statistical and mathematical techniques [19]. The various parameters and their interactions could be evaluated efficiently by this data analysis technology reducing the experimental group number [20, 21]. Uniform design underlined the uniformity of space filling in the experimental domain and the largest possible number of levels for each factor among all experimental designs [22]. UD can be simply used as a criterion to obtain better orthogonal designs.

The method in this study was developed to simultaneously determine sixteen flavonoids and phenolic acids of *Cuscuta chinensis* Lam. in different origins using liquid chromatography tandem mass-mass spectrum (HPLC-MS/MS) in the multiple reaction monitoring (MRM) acquisition mode for comprehensive quality control of *Cuscuta chinensis* Lam. To our knowledge, this is the first ever method identified to detect and quantify sixteen active components (Figure S1 in Supplementary Materials) of *Cuscuta chinensis* Lam. extracted via optimum conditions combined UD coupled with RSM and OD experiment. Moreover, the proposed method will be possible to lay the material basis for the evaluation and control of the quality of TCMs.

## 2. Experimental

### 2.1. Chemicals and Reagents

Four reference compounds including chlorogenic acid, caffeic acid, quercetin, and catechin were purchased from the National Institute for the Control of Pharmaceutical and Biological Products. Neochlorogenic acid, cryptochlorogenic acid, isochlorogenic acid A, isochlorogenic acid B, isochlorogenic acid C, *p*-coumaric acid, gallic acid, hyperin, isoquercitrin, campherol, rutin, isorhamnetin, astragalin, and apigenin were purchased from Chengdu Must Bio. Sci. and Chengdu Desite Bio-Technology Co., Ltd (Chengdu, China). Deionized water used for sample preparations and buffer solutions was purified by a Milli-Q Academic ultrapure water system (Millipore, Milford, MA, USA). Acetonitrile and methanol were purchased from Merck (Germany). Formic acid was purchased from Anaqua Chemicals Supply (ACS). All other chemicals were of analytical grade.

### 2.2. Herbal Plant

Various batches of *Cuscuta chinensis* Lam. were purchased from various provinces in China. The authenticity of *Cuscuta chinensis* Lam. species were identified by Professor Lin Ma (Tianjin University of Traditional Chinese Medicine), and the voucher specimens were deposited at Tianjin University of Traditional Chinese Medicine. The *Cuscuta chinensis* Lam. was smashed into powder using a pulverizer (Zhongcheng Pharmaceutical Machinery) and dried at 40°C. Then, the powders were passed over 50 meshes, which were prepared for the following tests.

### 2.3. Preparation of Standard Solutions and Samples

All standard solutions were individually dissolved in methanol at a stock concentration of 1 mg·mL^−1^. A series of mixed standard solutions were diluted with methanol in different concentrations. The stock solutions of catechin and gallic acid were also dissolved and diluted with methanol to a final concentration of 25 *µ*g·mL^−1^ and 1 *µ*g·mL^−1^, respectively. All of the solutions were stored at 4°C until analysis.

**Figure 1:**
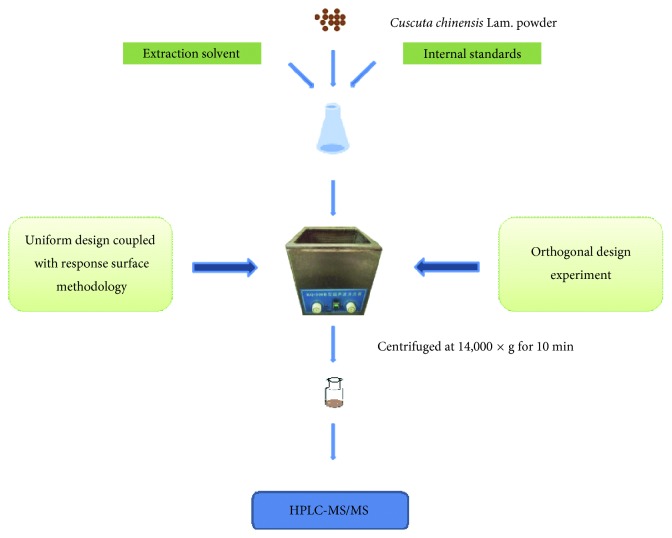
The schematic diagram of ultrasonic-assisted method coupled with HPLC-ESI-MS/MS.

Powdered sample (0.1 g) adding 10 *μ*L of 25 *µ*g·mL^−1^ catechin and 1 *µ*g·mL^−1^ gallic acid as internal standards were suspended in 45% ethanol-water (10 mL) and extracted in an ultrasonic bath for 120 min. Then, the sample was centrifuged at 14,000 ×g for 10 min. The supernatant was transferred and filtrated through a 0.22 *μ*m membrane prior to injection. 1 *μ*L of the solution was injected for analysis. The schematic diagram of the extraction method is shown in Figure 1.

### 2.4. Preparation of Quality Control Samples

Quality control (QC) samples of chlorogenic acid, caffeic acid, quercetin, neochlorogenic acid, cryptochlorogenic acid, isochlorogenic acid A, isochlorogenic acid B, isochlorogenic acid C, *p*-coumaric acid, hyperin, isoquercitrin, campherol, rutin, isorhamnetin, astragalin, and apigenin were prepared at low, medium, and high concentration levels by dissolving appropriate mixed standard solutions in methanol, respectively.

### 2.5. HPLC Condition and MS Condition

Qualitative analysis of the samples was performed using an Agilent HPLC 1200 system (Agilent Technologies, USA) coupled to an API 3200 triple quadrupole instrument (Agilent Corporation, CA, USA) with an electrospray ionization (ESI) source (Concord, Ontario, Canada). An Agilent Eclipse Plus C18 column (1.8 *μ*m, 4.6 mm × 150 mm) was equipped with a security guard Agilent C18 column (5 *μ*m, 2.1 mm × 12.5 mm). The mobile phase for the developed method consisted of acetonitrile (solvent A) and 0.05% aqueous formic acid in water (solvent B). The method employed a stepwise linear gradient as follows: 15%–19% solvent A at 0–3 min, 19%-20% solvent A at 3–9 min, 20%–30% solvent A at 9–12 min, 30%–48% solvent A at 12–12.5 min, 48%–52% solvent A at 12.5–14 min, 52%–54% solvent A at 14–17 min, 54%–60% solvent A at 17–18.5 min, and then 60%–81% solvent A at 18.5–20 min. The column was set at 35°C. In addition, the injection volume and low rate were 3 *μ*L and 3 mL·min^−1^, respectively.

The mass spectrometer was operated in the negative ion mode with curtain gas (CUR) of 45 psi, collision gas (CAD) of 5 psi, ion spray voltage (IS) of −4500 V, capillary temperature of 700°C, ion source gas 1 (GS1) of 40 psi, and ion source gas 2 (GS2) of 60 psi. The instrument was used in the tandem MS mode, by using the experiment of multiple reaction monitoring (MRM). A tandem mass spectrometry experiment that allows the selective isolation of the precursor ion in Q1, its subsequent fragmentation in a collision cell, and the final monitoring of a selected product ion in Q3 was done for the analysis of sixteen analytes and internal standards as shown in Table 1. The other parameters of eighteen compounds including declustering potential (DP), entrance potential (EP), collision energy (CE), collision cell exit potential (CXP), dwell time (DT), and retention time (RT) are also listed in Table 1.

## 3. Results and Discussion

### 3.1. Optimization of Extraction Procedure

#### 3.1.1. Optimization of Extraction by Uniform Design Coupled with Response Surface Methodology

As for the efficient extraction of active compounds including phenolic acids and flavonoids from *Cuscuta chinensis* Lam., some parameters which influenced the extraction efficiency were optimized. In this study, ethanol was chosen as the extraction solvent. In order to obtain the optimal extraction condition, the relationship among concentration of extraction solvent (*X*_1_), extraction time (*X*_2_), and ratio of liquid to solid (*X*_3_) was researched by uniform design (U12 (12 × 6 × 6)) (Table S1 in Supplementary Materials). The quadratic polynomial step-by-step regression method and data were analyzed by Uniform Design Version 3.00 software. A model was given as below to predict the response variables:

(1) $$\begin{array}{c} Y=b_{0}+b_{1}X_{1}+b_{2}X_{2}+b_{3}X_{3}+b_{1}^{2}X_{1}^{2}+b_{2}^{2}X_{2}^{2}+b_{2}^{3}X_{2}^{3}+b_{1}b_{2}X_{1}X_{2}+b_{1}b_{3}X_{1}X_{3}+b_{2}b_{3}X_{2}X_{3}, \end{array}$$

where *Y* is the predicted dependent variable; *b*_0_ is a constant that fixes the response at the central point of the experiment; *b*_1_, *b*_2_, and *b*_3_ are the regression coefficients for the linear effect terms; *b*_1_ *b*_2_, *b*_1_ *b*_3_, and *b*_2_ *b*_3_ are the interaction effect terms; and *b*_1_^2^, *b*_2_^2^, and *b*_3_^2^ are the quadratic effect terms, respectively [23]. Firstly, the extraction options for flavonoids (*Y*_1_) were statistically analyzed, and the predicted model developed for flavonoids (*Y*_1_) was as follows:

(2) $$\begin{array}{c} Y=2.07-2.82e^{-2}X_{1}-2.92e^{-2}X_{2}-4.27e^{-3}X_{3}+1.04e^{-4}X_{1}^{2}+1.93e^{-4}X_{1}X_{2}+2.48e^{-5}X_{1}X_{3}+1.16e^{-4}X_{2}^{2}+2.19e^{-5}X_{3}^{2}. \end{array}$$

**Figure 2:**
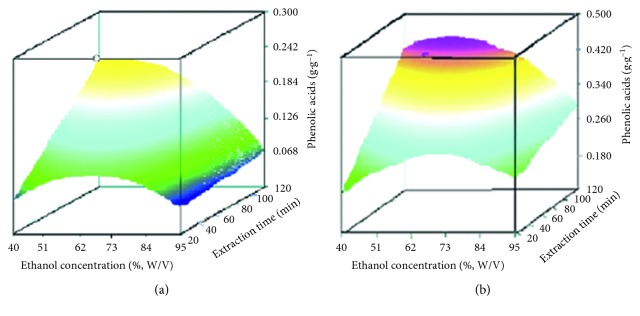
Effect of ethanol concentration and extraction time on the extraction efficiency of phenolic acids (a) and total analytes (b) in *Cuscuta chinensis* Lam.

According to the analysis of variance, the model with a good coefficient of determination (*R* = 0.95) was not significant (*F*-value 8.845, *P* > 0.05) which implied that the three factors (*X*_1_, *X*_2_, and *X*_3_) had no influence to the extraction efficiency of flavonoids. The extraction efficiency for phenolic acids was significantly influenced by the concentration of the extraction solvent (*P* < 0.05). On increasing the temperature from 20 to 120°C (45% ethanol), the phenolic acids content increased by about 0.106 g·g^−1^. In addition, on increasing the concentration of ethanol from 40% to 45%, the phenolic acids content increased by about 0.136 g·g^−1^. Further increasing the concentration of ethanol, the phenolic acids content was decreasing (Figure 2(a)). The extraction conditions for phenolic acids (*Y*_2_) were statistically analyzed and the predicted model established for phenolic acids (*Y*_2_) is shown as follows:

(3) $$\begin{array}{c} Y_{2}=-0.415+1.33e^{-2}X_{1}+2.58e^{-3}X_{2}+1.34e^{-3}X_{3}-7.57e^{-5}X_{1}^{2}-3.54e^{-5}X_{1}X_{2}-3.00e^{-5}X_{1}X_{3}-9.38e^{-6}\;X_{2}^{2}+1.90e^{-5}X_{2}X_{3}+1.40e^{-6}X_{3}^{2}. \end{array}$$

After the three revisions of the regression equation and eliminating nonsignificant items (*F*-value< *F*-critical value, *P* > 0.05), the predicted model established for phenolic acids (*Y*_2_) was modified as follows:

(4) $$\begin{array}{c} Y=-0.415+1.33e^{-2}X_{1}+2.58e^{-3}X_{3}+1.34e^{-3}x_{1}^{2}-7.57e^{-5}X_{1}X_{2}-3.54e^{-5}X_{1}X_{3}-3.00e^{-5}X_{2}X_{3}. \end{array}$$

On the basis of the elimination consequence (*X*_2_^2^), *X*_2_, and (*X*_3_^2^) were eliminated which indicated that the effect of extraction time (*X*_2_) and ratio of liquid to solid (*X*_3_) was not significant for the extraction for phenolic acids. Last but not the least, the effect of extraction parameters for total flavonoids and phenolic acids was conducted by RSM and developed a predicted model. The extraction efficiency for the total contents was influenced by the concentration of extraction solvent (*P* < 0.05). The total contents of flavonoids and phenolic acids ranged from 0.180 g·g^−1^ to 0.420 g·g^−1^, when the temperature increased from 20°C to 120°C (45% ethanol). In addition, there is a rising trend for the total contents of flavonoids and phenolic acids from 40% ethanol to 45% ethanol. Otherwise, the trend was changed when the concentration of ethanol declined continuously (Figure 2(b)). The predicted model established for total flavonoids and phenolic acids (*Y*_3_) is shown as follows:

(5) $$\begin{array}{c} Y=-1.96+4.49e^{-2}X_{1}+2.00e^{-2}X_{2}+3.28e^{-3}X_{3}-2.07e^{-4}X_{1}^{2}-1.69e^{-4}X_{1}X_{2}-7.81e^{-5}X_{1}X_{3}-8.18e^{-5}\;X_{2}^{2}+4.70e^{-5}X_{2}X_{3}. \end{array}$$

After quadruplicate revision of the regression equation and eliminating nonsignificant items (*F*-value< *F*-critical value, *P* > 0.05), the predicted model established for total flavonoids and phenolic acids (*Y*_3_) was modified as follows:

(6) $$\begin{array}{c} Y=-0.218+1.00e^{-2}X_{1}+1.24e^{-3}X_{3}-6.45e^{-5}X_{1}^{2}-1.54e^{-5}X_{1}X_{2}-2.43e^{-5}X_{1}X_{3}+1.77e^{-5}X_{2}X_{3}. \end{array}$$

The items including (*X*_2_^2^), *X*_2_, and *X*_3_ were eliminated (*F*-value< *F*-critical value, *P* > 0.05), which revealed that extraction time (*X*_2_) and ratio of liquid to solid (*X*_3_) were not significant factors in the extraction procedure.

Synthesizing the RSM analysis and the predicted model, 10 mL 45% ethanol-water and ultrasonic extraction for 120 min at room temperature were selected as the optimum extraction method.

#### 3.1.2. Optimization of Extraction by Orthogonal Design

After applying uniform design, which narrowed effectively the range of extraction conditions, some of the sophisticated tests should be investigated in succession by orthogonal design in order to obtain more efficient consequence. OD experiment was used to further optimize the extraction condition as an effective design method with small experimental range and less examined factors and levels. During this OD procedure, the experiment points for both training set and prediction set were concentrated in only a certain region derived from the results of UD coupled with RSM.

Firstly, the OD was designed to confirm the effects of various factors for extraction efficiency of flavonoids, including concentration of ethanol (A: 35% (A_1_), 40% (A_2_), 45% (A_3_)), extraction time (B: 80 min (B_1_), 100 min (B_2_), 120 min (B_3_)), ratio of sample to solvent (C: 100 g·mL^−1^ (C_1_), 120 g·mL^−1^ (C_2_), 125 g·mL^−1^(C_3_)), and D as the void item. When concentration of ethanol, extraction time, and ratio of sample to solvent was set to 45%, 120 min, and 125 g·mL^−1^, respectively, the best extraction efficiency could be obtained for analyzing flavonoids (Table S2 in Supplementary Materials). According to the result of the ANOVA test, *F*-value of extraction time exceeded *F*-critical values which meant this factor had significance in this extraction procedure. However, there was no significance in the other factors because their *F*-values were under *F*-critical value. Considering the above results and economy, 45%, 120 min, and 100 g·mL^−1^were chosen as concentration of ethanol, extraction time and ratio of sample to solvent for extracting flavonoids, respectively. Then, the following OD was carried out to investigate the effect of factors for extracting phenolic acids solely. The results displayed that the best extraction efficiency could be acquired for phenolic acids while concentration of ethanol, extraction time, and ratio of sample to solvent were set to 45%, 120 min, and 120 g·mL^−1^, respectively (Table S3 in Supplementary Materials). But, the ANOVA test of this OD showed that the ratio of sample to solvent in this part had no significance. Thus, best extraction factors for phenolic acids were chosen as 45% ethanol, 120 min of extraction time, and 120 g·mL^−1^ of sample to solvent. The last but not the least, further OD was applied to verify the optimum conditions for total phenolic acids and flavonoids. The results revealed that the extraction efficiency was best with 45% ethanol, 120 min of extraction time, and 100 g·mL^−1^ of sample to solvent (Table S4 in Supplementary Materials). The following ANOVA test also indicated that the concentration of ethanol and extraction time were significant factors, whereas the ratio of sample to solvent was not significant as the *F*-value was under the *F*-critical value. By comprehensive consideration of the cost of production and the extraction efficiency of phenolic acids and flavonoids, 100 g·mL^−1^ of sample to solvent, 45% ethanol-water, and ultrasonic extraction for 120 min at room temperature was selected as the optimum extraction method. This consequence revealed that the best pronounced condition could be obtained by OD experiment particularly and the optimum condition conducted by OD was the same with that obtained by UD.

### 3.2. Optimization of Chromatographic Conditions

#### 3.2.1. Optimization of HPLC Condition

To obtain good separation and stable peak retention value, the factors of the HPLC-ESI-MS/MS method was optimized in detail for analyzing the active compounds of *Cuscuta chinensis* Lam. The factors concerning the separation of sixteen components comprised the type of acid (formic acid, acetic acid, and ammonium formate), the concentration of acid (0.01%, 0.05%, and 0.1%), column temperature (25°C, 35°C, and 45°C), and flow rate (0.3 mL·min^−1^, 0.4 mL·min^−1^, and 0.5 mL·min^−1^). The effect of each factor was studied by orthogonal experiment design L9 (3^4^). Taking into account centered migration time and good peak response, hyperoside was selected as a reference to find the optimum HPLC condition, which is a main active component of *Cuscuta chinensis* Lam. The results (Table S5) showed that acetonitrile-0.05% formic acid aqueous solution in a step linear gradient, column temperature at 35°C, and 0.5 mL·min^−1^ flow rate should be finally set for the qualitative analysis of active compounds.

#### 3.2.2. Optimization of MS Condition

A tandem mass spectrometry approach was employed to obtain a quantitative determination of sixteen active markers and two internal standards. In order to increase the sensitivity of detection and improve the response value, optimization experiments of ion source parameters including CUR (30 psi, 35 psi, 40 psi, 45 psi), CAD (3 psi, 5 psi, 7 psi, 9 psi), IS (−2500 V, −3000 V, −3500 V, −4000 V, −4500 V), GS1 (25 psi, 30 psi, 35 psi, 40 psi, 45 psi), GS2 (30 psi, 40 psi, 50 psi, 60 psi), and TEM (300°C, 400°C, 500°C, 600°C, 700°C) were carried out. Consequently, the optimum ion source parameters were achieved when CUR was 45 psi, CAD was 5 psi, IS was −4500 V, GS1 was 40 psi, GS2 was 60 psi, and TEM was 700°C.

### 3.3. Selection of Internal Standard

In order to avoid the error caused by sample consistency and sample discrimination effect, internal standard should be added into the analytical sample solution. Considering the chemical property of analytical compounds and the interaction of retention time between analytes and internal standard, two kinds of compounds were selected as internal standard, covering catechin and gallic acid. Catechin and gallic acid were used as internal standards for the eight flavonoids and eight phenolic acids, respectively, because their chemical properties were similar with these two kinds of analytes and they would not impact the peaks of the targets in the chromatogram.

### 3.4. Method Validation

#### 3.4.1. Specificity

**Figure 3:**
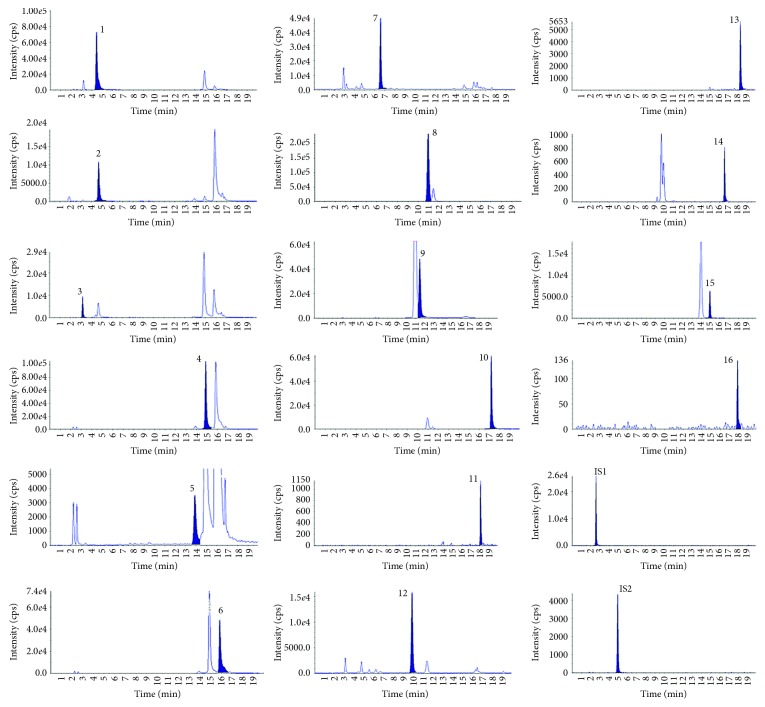
Typical chromatograms of LC–MS/MS for analytes and internal standards in *Cuscuta chinensis* Lam. 1, chlorogenic acid; 2, cryptochlorogenic acid; 3, neochlorogenic acid; 4, isochlorogenic acid A; 5, isochlorogenic acid B; 6, isochlorogenic acid C; 7, caffeic acid; 8, hyperin; 9, isoquercitrin; 10, quercetin; 11, campherol; 12, *p*-coumaric acid; 13, isorhamnetin; 14, rutin; 15, astragalin; 16, apigenin; IS1, catechin; IS 2, gallic acid.

Figure 3 shows the chromatograms of sixteen active components of *Cuscuta chinensis* Lam. in the MRM acquisition mode. The results of HPLC-MS/MS analysis of these markers demonstrated good shape of peaks, and no interfering peaks presented in the sample for analysis at the migration times of either analytes or internal standards.

#### 3.4.2. Linearity, Limits of Detection, and Repeatability

The calibration curve consisted of six concentration levels. The calibration graph was constructed by adding 10 *μ*L of 25 *µ*g·mL^−1^ catechin and 1 *µ*g·mL^−1^ gallic acid as internal standard. Sixteen plotted calibration curves and correlation coefficients (*r* > 0.999) confirmed that the curves were linear in the concentration ranges of each component. The limit of detection (LOD) and limit of quantification (LOQ) were considered as the concentrations of the compound that produced a signal-to-noise (S/N) ratio of 3 and 10, respectively. LODs and LOQs of each component ranged from 0.001 ng·mL^−1^ to 14 ng·mL^−1^ and 0.005 ng·mL^−1^ to 34.9 ng·mL^−1^, respectively (Table S6). The repeatability was evaluated by preparing the same sample with the same optimized extraction method (*n* = 6). The repeatability RSDs of sixteen targets were not more than 2.9%.

#### 3.4.3. Precision and Accuracy

Precision and accuracy were obtained by evaluating quality control samples containing sixteen active ingredients at low, medium, and high concentrations with respect to a calibration graph prepared each day (*n* = 6). The results of intraday and interday precision and accuracy are illustrated in Table S7. The accuracies of sixteen active components for both intraday and interday were within the range of 87.8%–120.7%. The RSDs for both intraday and interday were below 13.9%. These results indicated that the present method was accurate, reliable, and reproducible.

#### 3.4.4. Stability

The stability of sixteen active components at ambient temperature was assessed by analyzing quality control samples at low, medium, and high concentrations over 48 h storage at 4°C. The data of stability studies presented in Table S7 showed that RSDs of stability of sixteen active components were below 15.4%, when they were determined at 0 h, 2 h, 4 h, 6 h, 8 h, 12 h, 24 h, and 48 h, respectively. The results demonstrated that all of the components have good stability within 48 h of storage at the concentrations studied and this analytical method could be used to determine these compounds.

#### 3.4.5. Recovery

The recoveries were determined for the purpose of evaluation of precision and accuracy of the extraction method. The extraction recoveries of sixteen components were evaluated after extraction from *Cuscuta chinensis* Lam. to an equivalent amount of the standard solution including chlorogenic acid, cryptochlorogenic acid, neochlorogenic acid, isochlorogenic acid A, isochlorogenic acid B, isochlorogenic acid C, caffeic acid, hyperin, isoquercitrin, quercetin, campherol, *p*-coumaric acid, isorhamnetin, rutin, astragalin, and apigenin (*n* = 6). The mean recoveries of sixteen active components determined in the range of 89.9%–112.7% and RSDs were below 11.4% (Table S6). The results suggested that the extraction method showed good precision and accuracy.

### 3.5. Method Application

The proven ultrasonic-assisted extraction method coupled with HPLC-MS/MS method was applied to quantify sixteen active components in 31 batches of *Cuscuta chinensis* Lam. from different origins. The data of quantitative analysis illustrated that the total content of phenolic acids and flavonoids in *Cuscuta chinensis* Lam. from different origins varied significantly as shown in Tables 2 and 3. The total content of phenolic acids of *Cuscuta chinensis* Lam. from Chongqing province was relatively higher than others, which was the lowest from Yunnan. The highest total content of flavonoids of *Cuscuta chinensis* Lam. of these samples was determined from Henan province, and the lowest was assessed from Zhangshu.

## 4. Conclusion

In this research, an optical extraction condition was performed to get high yields of multiple active compositions by combining uniform design coupled with RSM and orthogonal design experiment. The HPLC-MS/MS procedure has been proposed to simultaneously determine sixteen active components present in different batches of *Cuscuta chinensis* Lam. with this effective extraction method. The developed method has been successfully applied to analyze the contents of phenolic acids and flavonoids using catechin and gallic acid as internal standards. The results demonstrated that this method offered excellent selectivity and sensitivity. Meanwhile, the use of HPLC-MS/MS could quantify phenolic acids and flavonoids and be successfully applied for quality control of *Cuscuta chinensis* Lam. in different origins. The contents of either phenolic acids or flavonoids of *Cuscuta chinensis* Lam. from the south of China were generally higher than those from the north of China. Consequently, the proposed HPLC-MS/MS method could be taken into consideration for future study of evaluation of quality control of TCMs.

## Figures and Tables

**Table 1:** Mass spectrometric parameters of analytical compounds and internal standards.

No.		Q1	Q3	DP (V)	EP (V)	CE (V)	CXP (V)	Acquisition time (second)	RT (min)
1	Chlorogenic acid	353.1	190.8	−24	−6	−33	−13	100	4.7
2	Cryptochlorogenic acid	353.2	173.0	−33	−5	−22	−2	100	4.96
3	Neochlorogenic acid	353.1	135.1	−24	−9	−46	−2	100	3.29
4	Isochlorogenic acid A	514.9	353.2	−64	−5	−28	−28	100	15.36
5	Isochlorogenic acid B	515.3	353.3	−67	−9	−28	−29	100	14.58
6	Isochlorogenic acid C	515.4	353.2	−51	−5	−21	−29	100	16.16
7	Caffeic acid	178.9	135.1	−33	−7	−23	−1	100	6.95
8	Hyperin	463.1	300.2	−71	−9	−42	−22	100	11.73
9	Isoquercitrin	463.2	300.0	−65	−8	−37	−25	100	12.28
10	Quercetin	301.2	151.2	−67	−8	−30	−2	100	17.25
11	Campherol	285.2	117.3	−80	−8	−56	−1	100	18.18
12	*p*-Coumaric acid	163.2	118.7	−36	−8	−23	−1	100	10.53
13	Isorhamnetin	315.3	300.2	−65	−10	−35	−25	100	18.34
14	Rutin	609.3	300.2	−28	−5	−46	−25	100	16.57
15	Astragalin	447.1	284.1	−65	5	−41	−22	100	15.29
16	Apigenin	269.2	117.1	−59	−9	−54	−1	100	17.9
IS1	Gallic acid	169.0	124.9	−32	−6	−24	−1	100	2.64
IS2	Catechin	289.1	123.0	−55	−9	−38	−2	100	4.94

**Table 2:** Contents of phenolic acids in samples from different origins (ng·g^−1^).

Collecting locations	Chlorogenic acid	Cryptochlorogenic acid	Neochlorogenic acid	Isochlorogenic acid A	Isochlorogenic acid B	Isochlorogenic acid C	Caffeic acid	Total phenolic acids
Qingdao	12000.0	1043.0	102.3	536.5	247.0	341.3	51.6	14321.7
Inner Mongolia	20700.0	2570.0	1021.3	3703.3	518.7	1416.7	88.1	30018.1
Ningxia	7060.0	652.3	97.4	260.7	—	417.7	82.0	8570.1
Beijing	567.0	361.0	138.7	—	—	—	—	1066.7
Suizhou	6966.7	736.7	91.6	119.0	—	358.7	134.7	8407.4
Zhangshu	343.3	175.0	65.0	63.6	—	285.7	—	932.6
Guizhou	20466.7	788.7	467.3	32633.3	1643.3	6716.7	213.3	62929.3
Yunnan	196.7	202.3	88.1	142.1	—	—	—	629.2
Henan	11900.0	501.7	141	187.0	375.0	689.0	119.0	13912.7
Anhui1	8710.0	423.5	101.1	675.0	288.7	398.7	127.5	10724.5
Tianjin	11633.3	—	—	149.5	266.0	410.5	—	12459.3
Xinjiang	9625.0	1640.0	178.5	212.0	321.3	781.7	141.0	12899.5
Jiangsu	603.3	—	143.0	—	—	—	—	746.3
Bozhou	21366.7	2060.0	211.0	857.0	411.0	757.0	125.7	25788.4
Anhui2	15233.3	1900	310.7	568.7	334.3	968.3	118.0	19433.3
Shanxi	5090.0	705.7	145.3	75.3	—	—	144.3	6160.6
Tangshan	143.5	—	522.5	—	—	—	—	666.0
Liaoning	5746.7	—	215.7	102.5	—	—	30.1	6095.0
Zhejiang	12500.0	1143.3	110.0	165.0	—	385.0	86.4	14389.7
Anhui3	13500.0	1466.7	208.0	256.7	—	330.3	105.7	15867.4
Jiangsu	20466.7	2010.0	312.7	1380.0	363.0	720.0	141.3	25393.7
Henan	2610.0	—	229.3	—	—	—	11.6	2850.9
Hebei	50500.0	5895.0	851.0	15150	1795.0	7005.0	2190.0	83386.0
Chifeng	23733.3	2730.0	1293.3	3953.3	585.3	1366.7	167.7	33829.6
Chongqing	30200.0	5100.0	1780.0	44633.3	3310.0	13300.0	3940.0	102263.3
Lingchuan	4995.0	633.3	120.3	453.0	—	487.5	70.1	6759.2
Shanxi	16033.3	1305.0	110.0	391.0	—	417.7	140.0	18397.0
Shanghai	4086.7	—	101.6	—	—	—	19.1	4207.4
Xuzhou	32266.7	3180.0	896.3	5056.7	618.3	1423.3	157.0	43598.3
Guangdong	15150.0	1550.0	165.9	—	—	—	100.2	16966.1
Qiyang	16333.3	605.3	256.3	2816.7	477.7	1090.0	295.0	21874.3

**Table 3:** Contents of flavonoids in samples from different origins (ng·g^−1^).

Collecting locations	Hyperin	Isoquercitrin	Quercetin	Campherol	*p*-Coumaric acid	Isorhamnetin	Rutin	Astragalin	Apigenin	Total flavonoids
Qingdao	19233.3	3240.0	49.3	3705.0	464.7	52.6	155.0	10266.7	1.1	37167.7
Inner Mongolia	34700.0	2276.7	60.1	451.0	479.3	49.6	119.0	926.3	0.8	39062.8
Ningxia	31833.3	1600.0	81.5	788.3	754.3	57.1	755.3	3270.0	0.9	39140.7
Beijing	3983.3	558.0	19.9	91.3	652.3	17.3	63.0	2270.0	1.0	7656.1
Suizhou	15933.3	3200.0	255.0	3316.7	779.3	123.0	51.1	7880.0	1.3	31539.7
Zhangshu	732.7	121.7	21.8	166.0	89.2	15.1	69.7	320.7	0.6	1537.5
Guizhou	3906.7	660.0	39.7	485.0	278.0	26.6	3583.3	1890.0	0.7	10870
Yunnan	2220.0	431.0	17.9	86.7	266.0	12.7	46.7	3020.0	0.7	6101.7
Henan	30166.7	5500.0	155.0	4113.3	1373.3	99.6	56.7	5416.7	1.5	46882.8
Anhui1	27000.0	3693.3	381.7	2356.7	906.0	129.7	201.3	4630.0	1.6	39300.3
Tianjin	20166.7	3533.3	119.0	3185.0	724.0	99.7	34.6	10295.0	1.0	38158.3
Xinjiang	12300.0	3380.0	78.8	5770.0	592.7	128.1	100.7	26900.0	1.0	49251.3
Jiangsu	6645.0	1226.7	20.0	186.0	690.7	16.1	96.0	6166.7	1.2	15048.4
Bozhou	23133.3	3723.3	44.2	4576.7	760.7	62.0	166.3	9186.7	1.2	41654.4
Anhui2	25433.3	4773.3	159.0	4066.7	504.7	143.3	48.0	19600.0	0.9	54729.2
Shanxi	13133.3	943.0	102.4	2273.3	637.0	89.3	431.0	4393.3	0.9	22003.5
Tangshan	880.0	209.0	21.9	196.7	959.0	15.3	29.9	2590.0	0.8	4902.6
Liaoning	13333.3	1400.0	19.6	112.5	571.0	13.8	286.7	3203.3	1.1	18941.3
Zhejiang	14133.3	2460.0	66.7	2800.0	551.7	65.2	56.6	6360.0	16.4	26509.9
Anhui3	20200.0	3676.7	54.5	4560.0	445.3	73.9	103.2	10346.7	1.2	39461.5
Jiangsu	29233.3	4030.0	106.5	2635.0	588.0	75.1	141.0	8493.3	1.2	45303.4
Henan	32250.0	6116.7	33.0	355.3	1153.3	17.3	322.7	29500.0	1.2	69749.5
Hebei	17666.7	807.0	18.9	431.3	1136.7	14.0	1316.7	1263.3	0.8	22655.4
Chifeng	24066.7	1510.0	58.1	98.6	359.0	30.1	117.0	134.3	0.7	26374.5
Chongqing	2326.7	472.0	20.1	426.3	856.7	18.5	4063.3	1500.0	2.5	9686.1
Lingchuan	6836.7	1130.0	38.1	1273.3	259.3	36.8	72.0	4990.0	0.7	14636.9
Shanxi	32066.7	4366.7	116.7	3610.0	718.0	81.9	52.1	5030.0	1.2	46043.3
Shanghai	25600.0	4180.0	19.5	256.0	458.7	21.7	68.1	5280.0	1.1	35885.1
Xuzhou	24400.0	1793.3	19.0	268.3	446.3	18.8	49.3	986.3	0.7	27982.0
Guangdong	7020.0	1206.7	14.0	1080.0	227.3	13.0	91.2	3116.7	0.7	12769.6
Qiyang	21666.7	3496.7	66.2	2190.0	590.3	44.5	531.3	4956.7	1.5	33543.9

## Data Availability

The data used to support the findings of this study are available from the corresponding author upon request.

## Supplementary Materials

Supplementary Materials Supplementary material contains revised articles on the experiment design for optimization of extraction of analytes from *Cuscuta chinensis* Lam. and the validation results for the analytical method. Table S1: uniform design (U_12_ (12 × 6 × 6)) for optimization of extraction of phenolic acids and flavonoids from *Cuscuta chinensis* Lam. Table S2: the results of orthogonal design [L_9_ (3^4^)] for optimizing of extraction of flavonoids from *Cuscuta chinensis* Lam. Table S3: the results of orthogonal design [L_9_ (3^4^)] for optimizing of extraction of phenolic acids from *Cuscuta chinensis* Lam. Table S4: the results of orthogonal design [L_9_ (3^4^)] for optimizing the extraction of flavonoids and phenolic acids from *Cuscuta chinensis* Lam. Table S5: the results of orthogonal design [L_9_ (3^4^)] for optimizing the HPLC conditions based on hyperoside of *Cuscuta chinensis* Lam. Table S6: the calibration curves, linearity ranges, LODs, LOQs, and recoveries of sixteen compounds in samples (*n* = 6). Table S7: intraday and interday accuracy and precision and stability of sixteen compounds (*n* = 6). Figure S1: the chemical structure of sixteen compounds and two internal standards.
