# Supplementary material for: Quantitative Analysis of Phenolic Acids and Flavonoids in Cuscuta chinensis Lam. by Synchronous Ultrasonic-Assisted Extraction with Response Surface Methodology
Source: J Anal Methods Chem. 2018 Dec 20;2018:6796720. doi: 10.1155/2018/6796720 (PMC6317117; doi:10.1155/2018/6796720)
Supplement: Supplementary Materials — Supplementary material contains revised articles on the experiment design for optimization of extraction of analytes from Cuscuta chinensis Lam. and the validation results for the analytical method. Table S1: uniform design (U12 (12 × 6 × 6)) for optimization of extraction of phenolic acids and flavonoids from Cuscuta chinensis Lam. Table S2: the results of orthogonal design [L9 (34)] for optimizing of extraction of flavonoids from Cuscuta chinensis Lam. Table S3: the results of orthogonal design [L9 (34)] for optimizing of extraction of phenolic acids from Cuscuta chinensis Lam. Table S4: the results of orthogonal design [L9 (34)] for optimizing the extraction of flavonoids and phenolic acids from Cuscuta chinensis Lam. Table S5: the results of orthogonal design [L9 (34)] for optimizing the HPLC conditions based on hyperoside of Cuscuta chinensis Lam. Table S6: the calibration curves, linearity ranges, LODs, LOQs, and recoveries of sixteen compounds in samples (n = 6). Table S7: intraday and interday accuracy and precision and stability of sixteen compounds (n = 6). Figure S1: the chemical structure of sixteen compounds and two internal standards. [file 6796720.f1.docx]

**Quantitative analysis of phenolic acids and flavonoids in *Cuscuta chinensis* Lam by** **synchronous ultrasonic-assisted extraction with response surface methodology**

Kunze Du^1,2^, Jin Li^1#^, Xinrong Guo^1,2^, Yuhong Li^1*^,Yan-xu Chang^1,2^*

^1^Tianjin State Key Laboratory of Modern Chinese Medicine, Tianjin University of Traditional Chinese Medicine, Tianjin, 300193, China;

^2^Tianjin Key Laboratory of Phytochemistry and Pharmaceutical Analysis, Tianjin University of Traditional Chinese Medicine, Tianjin, 300193, China

*Corresponding author:

Yan-xu Chang, Tianjin State Key Laboratory of Modern Chinese Medicine, Tianjin University of Traditional Chinese Medicine,

# The author contributs equally to first author in this study

E-mail: tcmcyx@126.com(Y-x chang); liyuhong@tjutcm.edu.cn(Y-h Li)

Tel.: +86-22-5959-6163; Fax: +86-22-5959-6163

Table S1. Uniform design（Ｕ12(12×6×6)）for optimization of extraction of phenolic acids and flavonoids from *Cuscuta chinensis Lam*

| No. | Concentration of ethanol (%） | Extraction time（min） | Ratio of sample to solvent（g mL^-1^） | Content of flavonoids（%） | Content of phenolic acids（%） | Total content（%） |
| --- | --- | --- | --- | --- | --- | --- |
| 1 | 40 | 60 | 100 | 0.133 | 0.173 | 0.306 |
| 2 | 45 | 120 | 60 | 0.130 | 0.156 | 0.287 |
| 3 | 50 | 60 | 20 | 0.119 | 0.095 | 0.213 |
| 4 | 55 | 120 | 100 | 0.199 | 0.243 | 0.442 |
| 5 | 60 | 40 | 60 | 0.128 | 0.145 | 0.273 |
| 6 | 65 | 100 | 20 | 0.126 | 0.093 | 0.219 |
| 7 | 70 | 40 | 120 | 0.184 | 0.155 | 0.339 |
| 8 | 75 | 100 | 80 | 0.201 | 0.139 | 0.341 |
| 9 | 80 | 20 | 40 | 0.207 | 0.131 | 0.338 |
| 10 | 85 | 80 | 120 | 0.201 | 0.136 | 0.337 |
| 11 | 90 | 20 | 80 | 0.150 | 0.086 | 0.236 |
| 12 | 95 | 80 | 40 | 0.165 | 0.050 | 0.215 |

Table S2.The results of orthogonal design [L_9_ (3^4^)] for optimizing of extraction of flavonoids from *Cuscuta chinensis* Lam

| Experiment No. | | A  (%) | | B  (min) | C  (g mL^-1^) | D | The total content (%) |  |
| --- | --- | --- | --- | --- | --- | --- | --- | --- |
| 1 | | | 1 | 1 | 1 | 1 | 1.25 |  |
| 2 | | | 1 | 2 | 2 | 2 | 1.00 |  |
| 3 | | | 1 | 3 | 3 | 3 | 1.32 |  |
| 4 | | | 2 | 1 | 2 | 3 | 1.19 |  |
| 5 | | | 2 | 2 | 3 | 1 | 1.15 |  |
| 6 | | | 2 | 3 | 1 | 2 | 1.26 |  |
| 7 | | | 3 | 1 | 3 | 2 | 1.32 |  |
| 8 | | | 3 | 2 | 1 | 3 | 1.23 |  |
| 9 | | | 3 | 3 | 2 | 1 | 1.31 |  |
| K_1_ ^a^ | | | 3.57 | 3.76 | 3.75 | 3.71 |  |  |
| K_2_ | | | 3.60 | 3.38 | 3.50 | 3.58 |  |  |
| K_3_ | | | 3.86 | 3.89 | 3.79 | 3.75 |  |  |
| R ^b^ | | | 0.09 | 0.17 | 0.09 | 0.06 |  |  |
| F-value^d^ | | | 1.227 | 3.592 | 1.171 | 0.406 |  |  |
| F critical value^d^ | 2.589 | | | 2.589 | 2.589 | 2.589 |  | |
| Significance |  | | | * |  |  |  | |

A is concentration of ethanol (%)

B is extraction time (min)

C is ratio of sample to solvent (g mL^-1^)

D is void item

^a^ The total content for each level between different factors . It is used to select the best level.

^b^ The range of content for different factors. It is used to rank the factors by their impacts on the content of all compounds.

^d^ ANOVA test value.

Table S3.The results of orthogonal design [L_9_ (3^4^)] for optimizing of extraction of phenolic acids from *Cuscuta chinensis* Lam

| Experiment No. | A  (%) | B  (min) | C  (g mL^-1^) | D | The total content  (%) |  |
| --- | --- | --- | --- | --- | --- | --- |
| 1 | 1 | 1 | 1 | 1 | 0.98 |  |
| 2 | 1 | 2 | 2 | 2 | 0.92 |  |
| 3 | 1 | 3 | 3 | 3 | 1.16 |  |
| 4 | 2 | 1 | 2 | 3 | 1.18 |  |
| 5 | 2 | 2 | 3 | 1 | 0.88 |  |
| 6 | 2 | 3 | 1 | 2 | 1.19 |  |
| 7 | 3 | 1 | 3 | 2 | 1.13 |  |
| 8 | 3 | 2 | 1 | 3 | 1.08 |  |
| 9 | 3 | 3 | 2 | 1 | 1.31 |  |
| K_1_ ^a^ | 3.05 | 3.29 | 3.25 | 3.17 |  | |
| K_2_ | 3.25 | 2.87 | 3.41 | 3.24 |  | |
| K_3_ | 3.52 | 3.66 | 3.17 | 3.41 |  |  |
| R ^b^ | 0.16 | 0.26 | 0.08 | 0.08 |  |  |
| F-value^d^ | 4.710 | 13.411 | 1.332 | 1.360 |  | |
| F critical value^d^ | 5.849 | 5.849 | 5.849 | 5.849 |  | |
| Significance | ** | *** |  |  |  | |

A is concentration of ethanol (%)

B is extraction time (min)

C is ratio of sample to solvent (g mL^-1^)

D is void item

^a^ The total content for each level between different factors . It is used to select the best level.

^b^ The range of content for different factors. It is used to rank the factors by their impacts on the content of all compounds.

^d^ ANOVA test value.

Table S4.The results of orthogonal design [L_9_ (3^4^)] for optimizing the extraction of flavonoids and phenolic acids from *Cuscuta chinensis* Lam

| Experiment No. | | A  (%) | B  (min) | C  (g mL^-1^) | D | The total content (%) | |
| --- | --- | --- | --- | --- | --- | --- | --- |
| 1 | | 1 | 1 | 1 | 1 | 2.23 | |
| 2 | | 1 | 2 | 2 | 2 | 1.92 | |
| 3 | | 1 | 3 | 3 | 3 | 2.48 | |
| 4 | | 2 | 1 | 2 | 3 | 2.37 | |
| 5 | | 2 | 2 | 3 | 1 | 2.02 | |
| 6 | | 2 | 3 | 1 | 2 | 2.46 | |
| 7 | | 3 | 1 | 3 | 2 | 2.45 | |
| 8 | | 3 | 2 | 1 | 3 | 2.31 | |
| 9 | 3 | 3 | 2 | 1 | 2.62 | |  |
| K_1_ ^a^ | | 6.63 | 7.05 | 6.99 | 6.87 |  |  |
| K_2_ | | 6.85 | 6.25 | 6.91 | 6.82 |  |  |
| K_3_ | | 7.38 | 7.56 | 6.95 | 7.16 |  |  |
| R ^b^ | | 0.25 | 0.44 | 0.03 | 0.11 |  |  |
| F-value^d^ | | 3.157 | 9.226 | 0.034 | 0.721 |  |  |
| F critical value^d^ | | 5.849 | 5.849 | 5.849 | 5.849 |  |  |
| Significance | | * | *** |  |  |  |  |

A is concentration of ethanol (%)

B is extraction time (min)

C is ratio of sample to solvent (g mL^-1^)

D is void item

^a^ The total content for each level between different factors . It is used to select the best level.

^b^ The range of content for different factors. It is used to rank the factors by their impacts on the content of all compounds.

^d^ ANOVA test value.

Table S5. The results of orthogonal design [L_9_ (3^4^)] for optimizing the HPLC conditions based on hyperoside of *Cuscuta chinensis* Lam

| Experiment No. | Conditions | | | | Response |
| --- | --- | --- | --- | --- | --- |
|  | The concentration of acid (%) | Column temperature(^◦^C) | Flow rate  (mL min^-1^) | The type of acid |  |
| 1 | 0.01 | 25 | 0.3 | formic acid | 4.7e^5^ |
| 2 | 0.01 | 35 | 0.4 | acetic acid | 3.5e^5^ |
| 3 | 0.01 | 45 | 0.5 | [ammonium](javascript:void(0);) [formate](javascript:void(0);) | 5.2e^5^ |
| 4 | 0.05 | 25 | 0.4 | [ammonium](javascript:void(0);) [formate](javascript:void(0);) | 3.3e^5^ |
| 5 | 0.05 | 35 | 0.5 | formic acid | 5.8e^5^ |
| 6 | 0.05 | 45 | 0.3 | acetic acid | 2.8e^5^ |
| 7 | 0.1 | 25 | 0.5 | acetic acid | 5.6e^5^ |
| 8 | 0.1 | 35 | 0.3 | [ammonium](javascript:void(0);) [formate](javascript:void(0);) | 5.7e^4^ |
| 9 | 0.1 | 45 | 0.4 | formic acid | 3.4e^5^ |

Table S6. The calibration curves, linearity ranges, LODs, LOQs and recoveries of sixteen compounds in samples (n=6)

| Compounds | Calibration curves | R | LOD  (μg/mL) | LOQ  (μg/mL) | Linearity range (μg/mL) | Recovery | | Repeatability | | |
| --- | --- | --- | --- | --- | --- | --- | --- | --- | --- | --- |
|  |  |  |  |  |  | Average (%) | RSD (%) | | RSD (%) | |
| [chlorogenic](javascript:void(0);) [acid](javascript:void(0);) | Y=0.00424x-0.0886 | 0.9990 | 3.3 | 16.6 | 83.2-41600 | 99.8 | 6.1 | | 2.7 |  |
| cryptochlorogenic acid | Y=0.00175x-0.0134 | 0.9992 | 13.3 | 33.3 | 166 -16600 | 99.2 | 9.7 | | 1.4 |  |
| neochlorogenic acid | Y=0.00195x+0.0133 | 0.9995 | 14 | 34.9 | 174-17400 | 107.4 | 9.2 | | 2.4 |  |
| isochlorogenic acid A | Y=0.00154x-0.0727 | 0.9990 | 0.1 | 0.3 | 158-39600 | 101.5 | 6.0 | | 0.8 |  |
| isochlorogenic acid B | Y=0.00111x-0.0159 | 0.9990 | 12.4 | 31.0 | 62.1-15500 | 101.8 | 7.2 | | 2.8 |  |
| isochlorogenic acid C | Y=0.00118x+0.000289 | 0.9998 | 12.9 | 32.3 | 32.3-8080 | 89.9 | 1.4 | | 2.9 |  |
| caffeic acid | Y=0.0144x+0.0301 | 0.9994 | 3.2 | 8 | 40-4000 | 97.1 | 0.8 | | 2.7 |  |
| hyperin | Y=0.0028x+0.427 | 0.9991 | 13.4 | 26.9 | 336-84000 | 103 | 2.8 | | 1.2 |  |
| isoquercitrin | Y=0.00427-0.15 | 0.9995 | 3.4 | 6.7 | 168-2100 | 99.4 | 6.3 | | 2.9 |  |
| quercetin | Y=0.0108x+0.184 | 0.9994 | 0.03 | 0.1 | 16.8-8400 | 92.2 | 3.2 | | 2.6 |  |
| campherol | Y=0.000658x+0.0173 | 0.9999 | 0.1 | 0.5 | 67.2-16800 | 98.3 | 7.6 | | 2.9 |  |
| p-coumaric acid | Y=0.00482x-0.156 | 0.9991 | 3.2 | 6.5 | 162-20200 | 90.4 | 3.4 | | 2.5 |  |
| isorhamnetin | Y=0.012x+0.0598 | 0.9992 | 0.03 | 0.13 | 16.2-4040 | 102 | 9.3 | | 2.6 |  |
| rutin | Y=0.000806x+0.118 | 0.9993 | 6.8 | 13.7 | 171-42800 | 110.7 | 11.4 | | 1.9 |  |
| astragalin | Y=0.00345x+0.0663 | 0.9994 | 0.1 | 0.3 | 32.6-20400 | 99.9 | 9.8 | | 2.2 |  |
| apigenin | Y=0.0474x+0.0207 | 0.9996 | 0.001 | 0.005 | 0.28-176 | 112.7 | 9.8 | | 2.2 |  |

Table S7 Intra-day and Inter-day accuracy and precision, stability of sixteen compounds (n = 6)

| Compounds | Concentrations  (ng·mL^-1^) | Intra-day precision | | Inter-day  precision |  | Stability (%) |
| --- | --- | --- | --- | --- | --- | --- |
|  |  | Accuracy (%) | RSD (%) | Accuracy  (%) | RSD (%) |  |
| [chlorogenic](javascript:void(0);) [acid](javascript:void(0);) | 416.0 | 105.8 | 3.7 | 111.0 | 5.5 | 5.8 |
|  | 2080.0 | 112.0 | 4.3 | 116.0 | 7.1 | 10.1 |
|  | 10400.0 | 111.3 | 3.9 | 120.7 | 7.6 | 8.2 |
| cryptochlorogenic acid | 166.4 | 96.4 | 13.9 | 102.6 | 7.1 | 13.8 |
|  | 832.0 | 112.3 | 8.9 | 105.4 | 3.3 | 7.8 |
|  | 4160.0 | 110.0 | 6.6 | 115.6 | 7.9 | 6.7 |
| neochlorogenic acid | 174.4 | 113.5 | 7.9 | 115.6 | 7.3 | 8.5 |
|  | 872.0 | 106.4 | 4.3 | 107.3 | 5.4 | 9.3 |
|  | 4360.0 | 111.1 | 5.6 | 113.6 | 6.8 | 7.3 |
| isochlorogenic acid A | 396.0 | 115.6 | 4.7 | 114.8 | 7.6 | 11.0 |
|  | 1980.0 | 106.7 | 10.5 | 108.2 | 3.2 | 9.3 |
|  | 9900.0 | 104.5 | 7.0 | 114.9 | 2.2 | 10.2 |
| isochlorogenic acid B | 155.2 | 96.5 | 3.2 | 104.0 | 4.9 | 11.3 |
|  | 776.0 | 102.8 | 11.1 | 108.9 | 5.6 | 5.7 |
|  | 3880.0 | 108.3 | 3.6 | 115.9 | 4.0 | 13.5 |
| isochlorogenic acid C | 161.6 | 117.7 | 9.5 | 110.9 | 7.1 | 8.0 |
|  | 808.0 | 107.1 | 3.1 | 111.7 | 4.2 | 6.8 |
|  | 4040.0 | 112.6 | 1.5 | 114.3 | 5.2 | 10.9 |
| caffeic acid | 40.0 | 113.3 | 4.2 | 118.6 | 6.2 | 15.4 |
|  | 200.0 | 110.2 | 4.3 | 119.0 | 4.1 | 7.8 |
|  | 1000.0 | 110.8 | 6.5 | 115.0 | 4.2 | 8.9 |
| hyperin | 1680.0 | 113.9 | 3.6 | 101.9 | 2.3 | 5.1 |
|  | 8400.0 | 102.3 | 5.7 | 102.2 | 2.9 | 8.4 |
|  | 42000.0 | 102.4 | 7.4 | 101.8 | 4.8 | 14.2 |
| isoquercitrin | 420.0 | 102.6 | 4.2 | 93.9 | 3.0 | 8.3 |
|  | 2100.0 | 90.7 | 3.0 | 87.8 | 2.5 | 8.6 |
|  | 10500.0 | 97.4 | 8.4 | 101.3 | 5.5 | 12.5 |
| quercetin | 84.0 | 111.3 | 5.2 | 115.0 | 7.9 | 14.4 |
|  | 420.0 | 102.3 | 7.8 | 107.0 | 4.0 | 11.2 |
|  | 2100.0 | 109.9 | 4.6 | 109.0 | 1.0 | 14.1 |
| campherol | 336.0 | 101.9 | 3.0 | 107.6 | 5.2 | 7.8 |
|  | 1680.0 | 104.7 | 5.1 | 109.6 | 2.5 | 7.5 |
|  | 8400.0 | 101.5 | 4.3 | 108.2 | 5.3 | 10.1 |
|  | 404.0 | 96.2 | 5.7 | 107.9 | 3.0 | 6.4 |
| p-coumaric acid | 2020.0 | 97.1 | 4.5 | 101.5 | 2.0 | 7.1 |
|  | 10100.0 | 98.0 | 8.1 | 101.0 | 5.2 | 11.9 |
| isorhamnetin | 80.8 | 107.9 | 7.0 | 99.0 | 2.0 | 13.2 |
|  | 404.0 | 90.8 | 6.4 | 97.0 | 2.8 | 5.6 |
|  | 2020.0 | 95.4 | 8.2 | 99.8 | 5.6 | 10.2 |
| rutin | 856.0 | 108.8 | 4.7 | 103.3 | 3.2 | 5.4 |
|  | 4280.0 | 102.1 | 11.9 | 100.9 | 4.4 | 11.4 |
|  | 21400.0 | 98.7 | 9.1 | 97.3 | 5.4 | 16.0 |
| astragalin | 408.0 | 110.8 | 12.1 | 104.3 | 5.2 | 7.8 |
|  | 2040.0 | 103.8 | 3.8 | 105.2 | 2.2 | 9.5 |
|  | 10200.0 | 101.4 | 6.4 | 104.9 | 6.6 | 11.8 |
| apigenin | 3.5 | 94.7 | 8.7 | 104.8 | 11.9 | 9.7 |
|  | 17.6 | 88.6 | 5.0 | 96.3 | 2.1 | 7.3 |
|  | 88.0 | 92.4 | 3.7 | 101.4 | 9.0 | 7.0 |

[chlorogenic](javascript:void(0);) [acid](javascript:void(0);) cryptochlorogenic acid neochlorogenic acid

isochlorogenic acid A isochlorogenic acid B isochlorogenic acid C

hyperin isoquercitrin quercetin

campherol p-coumaric acid isorhamnetin

rutin astragalin apigenin

caffeic acid gallic acid catechin

Figure S1. The chemical structure of sixteen compounds and two internal standards
